# Two new species of *Haploporus* (Polyporales, Basidiomycota) from China and Ecuador based on morphology and phylogeny

**DOI:** 10.3389/fcimb.2023.1133839

**Published:** 2023-02-21

**Authors:** Xiao-Wu Man, Yu-Cheng Dai, Lu-Sen Bian, Meng Zhou, Heng Zhao, Josef Vlasák

**Affiliations:** ^1^ Institute of Microbiology, School of Ecology and Nature Conservation, Beijing Forestry University, Beijing, China; ^2^ Experimental Centre of Forestry in North China, Warm Temperate Zone Forestry Jiulong Mountain National Permanent Scientific Research Base, Chinese Academy of Forestry, Beijing, China; ^3^ Inst. Plant Mol. Biol., Biology Centre of the Academy of Sciences of the Czech Republic, České Budějovice, Czechia

**Keywords:** polyporaceae, wood-rotting fungi, taxonomy, fungi diversity, new taxa

## Abstract

At present, 25 species are accepted in *Haploporus* and are distributed in Asia, Europe, North America, South America, Australia, and Africa. In this study, two new species, *Haploporus ecuadorensis* from Ecuador and *H. monomitica* from China, are described and illustrated based on morphological examination and phylogenetic analyses. *H. ecuadorensis* is characterized by annual, resupinate basidiomata with pinkish buff to honey yellow hymenophore when dry, round to angular pores of 2–4 per mm, a dimitic hyphal structure with generative hyphae bearing clamp connections, hyphae at dissepiment edge usually with one or two simple septa, the presence of dendrohyphidia and cystidioles, and oblong to ellipsoid basidiospores measuring 14.9–17.9 × 6.9–8.8 µm. *Haploporus monomitica* differs from other *Haploporus* species in that it has a monomitic hyphal system and strongly dextrinoid basidiospores. The differences between the new species and morphologically similar and phylogenetically related species are discussed. In addition, an updated key to 27 species of *Haploporus* is provided.

## Introduction

The genus *Haploporus* Bondartsev & Singer, belonging to Polyporaceae, Polyporales, Agaricomycetes, and Basidiomycota, was established by A. S. Bondartsev and R. Singer in 1944 and typified by *Haploporus odorus* (Sommerf.) Bondartsev & Singer (Singer, 1944). It is characterized by annual to perennial, resupinate to pileate basidiomata, a dimitic to trimitic hyphal system with clamp connections on the generative hyphae, cyanophilous skeletal hyphae, and thick-walled, cyanophilous, and ornamented basidiospores, causing a white rot of wood (Singer, 1944; Dai et al., 2002; Piatek, 2005; Li et al., 2007; Shen et al., 2016; Zhou et al., 2019; Zhou et al., 2021; Wu et al., 2022a).

In 1963, F. Kotlaba and Z. Pouzar proposed the genus *Pachykytospora* Kotl. & Pouzar (Kotlaba & Pouzar, 1963). However, most species of *Pachykytospora*, including *P. alabamae* (Berk. & Cooke) Ryvarden, *P. nanospora* A. David & Rajchenb, *P. nepalensis* T. Hatt., *P. papyracea* (Cooke) Ryvarden, *P. thindii* Natarajan & Koland, and *P. tuberculosa* (Fr.) Kotl. & Pouzar, have been transferred to *Haploporus* according to morphological characteristics and molecular phylogenetic analyses (Dai & Li, 2002; Piatek, 2003; Piatek, 2005; Shen et al., 2016; Zhou et al., 2021).

The genus *Haploporus* has been extensively studied in Australia, Brazil, China, Kenya, Sri Lanka, Sweden, and the USA (Lira et al., 2018; Zhou et al., 2019; Decock et al., 2021; Zhou et al., 2021). In the last decade, 16 species were described or combined in *Haploporus*, namely, *H. angustisporus* Meng Zhou & Y.C. Dai; *H. bicolor* Y.C. Dai, Meng Zhou, & Yuan; *H. brasiliensis* Nogueira-Melo & Ryvarden; *H. crassus* Meng Zhou & Y.C. Dai; *H. cylindrosporus* L.L. Shen, Y.C. Dai, & B.K. Cui; *H. eichelbaumii* (Henn.) Decock; *H. gilbertsonii* Meng Zhou, Vlasák, & Y.C. Dai; *H. grandisporus* Decock; *H. longisporus* Y.C. Dai, Meng Zhou, & Vlasák; *H. microsporus* L.L. Shen, Y.C. Dai, & B.K. Cui; *H. pileatus* Ryvarden; *H. pirongia* (G. Cunn.) Meng Zhou, Y.C. Dai, & T.W. May; *H. punctatus* Y.C. Dai, Meng Zhou, & Yuan; *H. septatus* L.L. Shen, Y.C. Dai, & B.K. Cui; *H. srilankensis* Y.C. Dai, Meng Zhou, & Yuan; and *H. subpapyraceus* L.L. Shen, Y.C. Dai, & B.K. Cui (Shen et al., 2016; Lira et al., 2018; Zhou et al., 2019; Decock et al., 2021; Zhou et al., 2021). Prior to our work, a total of 25 species was accepted in the genus (Dai et al., 2002; Hattori et al., 2002; Piatek, 2005; Li et al., 2007; Dai & Kashiwadani, 2009; Shen et al., 2016; Lira et al., 2018; Zhou et al., 2019; Decock et al., 2021; Zhou et al., 2021).

During a study on polypores from Ecuador and China, we collected specimens that morphologically fit the definition of *Haploporus*. After further examination and phylogenetic analysis, they formed two distinct lineages within *Haploporus*, and are morphologically different from the existing species in the genus. Thus, we describe them here as two new species.

## Materials and methods

### Morphological studies

The studied *Haploporus* specimens are deposited in the herbarium of the Institute of Microbiology, Beijing Forestry University (BJFC), the private herbarium of Josef Vlasák (JV), and the National Museum Prague of Czech Republic (PRM). For the morphological description, we followed the method from a previous study (Wu et al., 2022b). Color terms are from Anonymous (1969) and Petersen (1996).

### DNA extraction, PCR, and sequencing

The DNA was extracted from the dried specimens using a rapid plant genome extraction kit (Aidlab Biotechnologies Co., Ltd, Beijing, China), following the manufacturer’s protocol. The internal transcribed spacers (ITS), large subunit of nuclear ribosomal RNA gene (LSU), and small subunit mitochondrial rRNA gene (mtSSU) were amplified with primer pairs ITS 5 (5′‐GGA AGT AAA AGT CGT AAC AAG G‐3′) and ITS 4 (5′‐TCC TCC GCT TAT TGATAT GC‐3′; White et al., 1990), LR0R (5′‐ACC CGC TGA ACT TAA GC‐3′) and LR7 (5′‐TAC TAC CAC CAA GAT CT‐3′; http://www.biology.duke.edu/fungi/mycolab/primers.htm ), and MS1 (5′‐CAG CAG TCA AGA ATA TTA GTC AAT G‐3′) and MS2 5′‐GCG GAT TAT CGA ATT AAA TAA C‐3′; White et al., 1990), respectively. The PCR procedures were as follows: for ITS and mtSSU regions, an initial denaturation at 95°C for 3 min, followed by 34 cycles at 94°C for 40 s, 54°C for ITS and 55°C for mtSSU for 45 s and 72°C for 1 min, and a final extension of 72°C for 10 min; for the LSU region, an initial denaturation at 94°C for 1 min, followed by 34 cycles at 94°C for 30 s, 50°C for 1 min and 72°C for 1.5 min, and a final extension of 72°C for 10 min (Zhou et al., 2021; Zhao et al., 2022c). The PCR products were sequenced using BGI Tech Solutions (Beijing Liuhe Co., Ltd., Beijing, China). Finally, all the new sequences were submitted to GenBank, and the accession numbers are shown in **Table 1**.

**Table 1:** Taxa information and GenBank accession numbers used in this study.

Species	Sample no.	GenBank Accession no.			Country
		ITS	LSU	mt-SSU	
*Haploporus alabamae*	Dollinger 895	KY264038	MK433606	MW463004	USA
*H. alabamae*	JV 1704/75	MK429754	MK433607	MW463005	Costa Rica
*H. angustisporus*	Dai 10951	KX900634	KX900681	MW463006	China
*H. bicolor*	Dai 19951	MW465684	MW462995	–	China
*H. crassus*	Dai 13580	MW465669	KU941865	–	China
*H. cylindrosporus*	Dai 15664	KU941854	KU941878	KU941903	China
***H. ecuadorensis***	**JV1906/C10-J**	**MW465661**	**OP948227**	**OP948226**	**Ecuador**
*H. eichelbaumii*	Congo 1	MT758256	MT758256	–	Congo
*H. eichelbaumii*	KE-17-238	MT758261	MT758261	–	Kenya
*H. gilbertsonii*	JV 1611/5-J	MK429756	MK433609	MW463007	USA
*H. grandisporus*	KE-16-130	MT758242	MT758242	–	Kenya
*H. grandisporus*	KE-17-228	MT758244	MT758244	–	Kenya
*H. latisporus*	Dai 11873	KU941847	KU941871	MW463008	China
*H. longisporus*	JV 1906/C11-J	MW465685	MW462996	–	Ecuador
*H. microsporus*	Dai 12147	KU941861	KU941885	–	China
***H. monomitica***	**Dai 24429**	**OP725709**	**OP725712**	**–**	**China**
***H. monomitica***	**Dai 24446**	**OP725710**	**OP725713**	**OP725715**	**China**
***H. monomitica***	**Dai 24451**	**OP725711**	**OP725714**	**OP725716**	**China**
*H. nanosporus*	MUCL 47447	MT782648	MT777438	–	Gabon
*H. nanosporus*	MUCL 47559	MT782650	MT777440	–	Gabon
*H. nepalensis*	Dai 12937	KU941855	KU941879	KU941904	China
*H. odorus*	Dai 11296	KU941845	KU941869	KU941894	China
*H. odorus*	Yuan 2365	KU941846	KU941870	KU941895	China
*H. papyraceus*	Dai 10778	KU941839	KU941863	KU941888	China
*H. pirongia*	Dai 18659	MH631017	MH631021	MW463009	Australia
*H. punctatus*	Dai19628	MW465687	MW462998	MW463011	Sri Lanka
*H. septatus*	Cui 4100	KU941844	KU941868	KU941893	China
*H. srilankensis*	Dai19523	MW465688	MW462999	MW463012	Sri Lanka
*H. subpapyraceus*	Cui 2651	KU941842	KU941866	KU941891	China
*H. subpapyraceus*	Dai 9324	KU941841	KU941865	KU941890	China
*H. subtrameteus*	KUC20121102-36	KJ668536	KJ668389	–	Korea
*Haploporus* sp. 1	LR11231	MT758249	MT758249	–	Malawi
*H. thindii*	Cui 9373	KU941851	KU941875	KU941900	China
*H. thindii*	Cui 9682	KU941852	KU941876	KU941901	China
*H. tuberculosus*	15559	KU941857	KU941881	KU941906	Sweden
*Perenniporia hainaniana*	Cui 6364	JQ861743	JQ861759	KF051044	China
*P. medulla-panis*	Cui 3274	JN112792	JN112793	KF051043	China

The sequences generated in this study are in bold. “−” represents sequences unavailable in GenBank.

### Phylogenetic analyses

The sequences generated were aligned with sequences downloaded from GenBank (**Table 1**) using MAFFT (version 7) and then manually adjusted (Katoh & Standley, 2013). A dataset of 34 specimens consisting of ITS, LSU, and mtSSU sequences was analyzed using Maximum Likelihood (ML), Maximum Parsimony (MP), and Bayesian Inference (BI) phylogenetic analyses using RAxML (version 8; Stamatakis, 2014), PAUP (version 4.0b10; Swofford, 2002), and MrBayes (version 3.2.7a; Ronquist et al., 2012), respectively, following Zhao et al, 2021; Zhao et al, 2022a; Zhao et al, 2022b). The ModelTest-NG (version 0.1.7; Darriba et al., 2020) determined the best models of ITS, LSU, and mtSSU sequences. The ML analysis was carried out with 1,000 bootstrap replications using the GTR + I + G substitution model. The MP analysis was conducted using 1,000 bootstrap replications with the heuristic search option. The BI analysis was performed for two million generations with random initial trees, using the GTR + I + G substitution model and the first 25% were set as burn-in.

The phylogenetic tree was visualized using FigTree version 1.4.4 (http://tree.bio.ed.ac.uk/software/figtree/ ). Branches that received bootstrap support for ML, BP, and Bayesian Posterior Probabilities (BPP) greater than or equal to 50% (ML/BP) and 0.95 (BPP) were considered as significantly supported, respectively.

## Results

### Phylogeny

**Figure 1:**
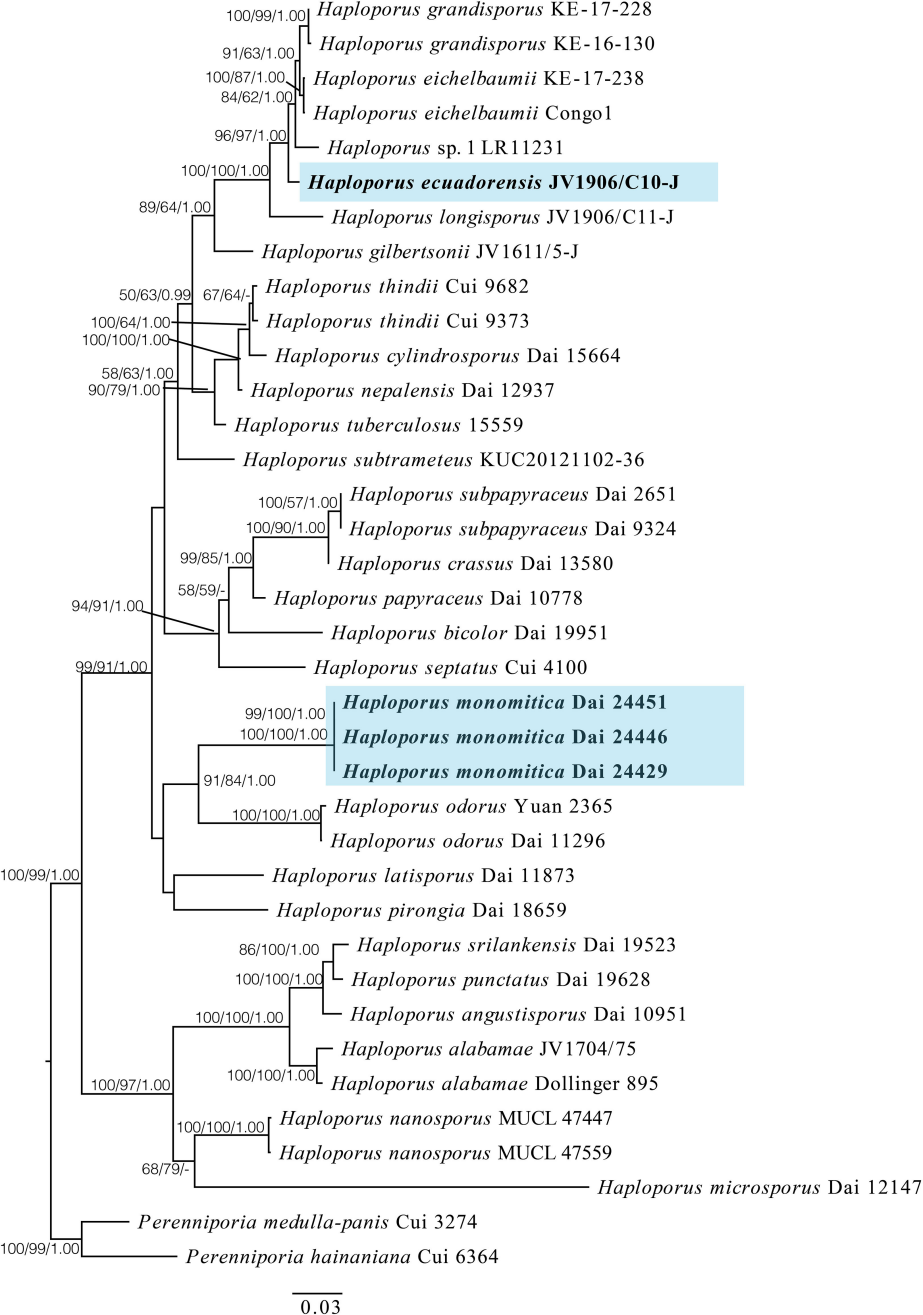
A maximum likelihood phylogenetic tree of *Haploporus* based on ITS, LSU, and mtSSU sequences, with two specimens of *Perenniporia hainaniana* and *P. medulla-panis* used as outgroups. The new species *Haploporus ecuadorensis* and *H. monomitica* are shaded. Maximum likelihood bootstrap values (≥50%)/maximum parsimony bootstrap values (≥50%)/Bayesian posterior probabilities (≥0.95) of each clade are indicated along branches. A scale bar below indicates the number of substitutions per site.

In this study, the combined ITS + LSU + mtSSU dataset included sequences from 37 specimens, representing 25 species of *Haploporus* and 2 species of *Perenniporia* Murrill as the outgroup (**Table 1** and **Figure 1**). The aligned dataset had a length of 1,932 characters, of which 540 were constant characters, 122 were parsimony-uninformative characters, and 221 were parsimony-informative characters. The MP analysis yielded a tree with a length of 812, a consistency index of 0.5246, a homoplasy index of 0.4754, a retention index of 0.7551, and a rescaled consistency index of 0.3961. The best model for the ITS + LSU + mtSSU aligned dataset was GTR + I + G in the Bayesian analysis, and the average standard deviation of split frequencies is 0.00424. The phylograms of Bayesian analysis, MP analysis, and ML analysis are similar in topology, and the ML tree was chosen to represent the phylogenetic relationships (**Figure 1**).

The phylogenetic tree suggests that the specimen of *H. ecuadorensis* forms an independent lineage in the *Haploporus* clade, and specimens of *H. monomitica* are closely related to *H. odorus* with strong support.

### Taxonomy

**Figure 2:**
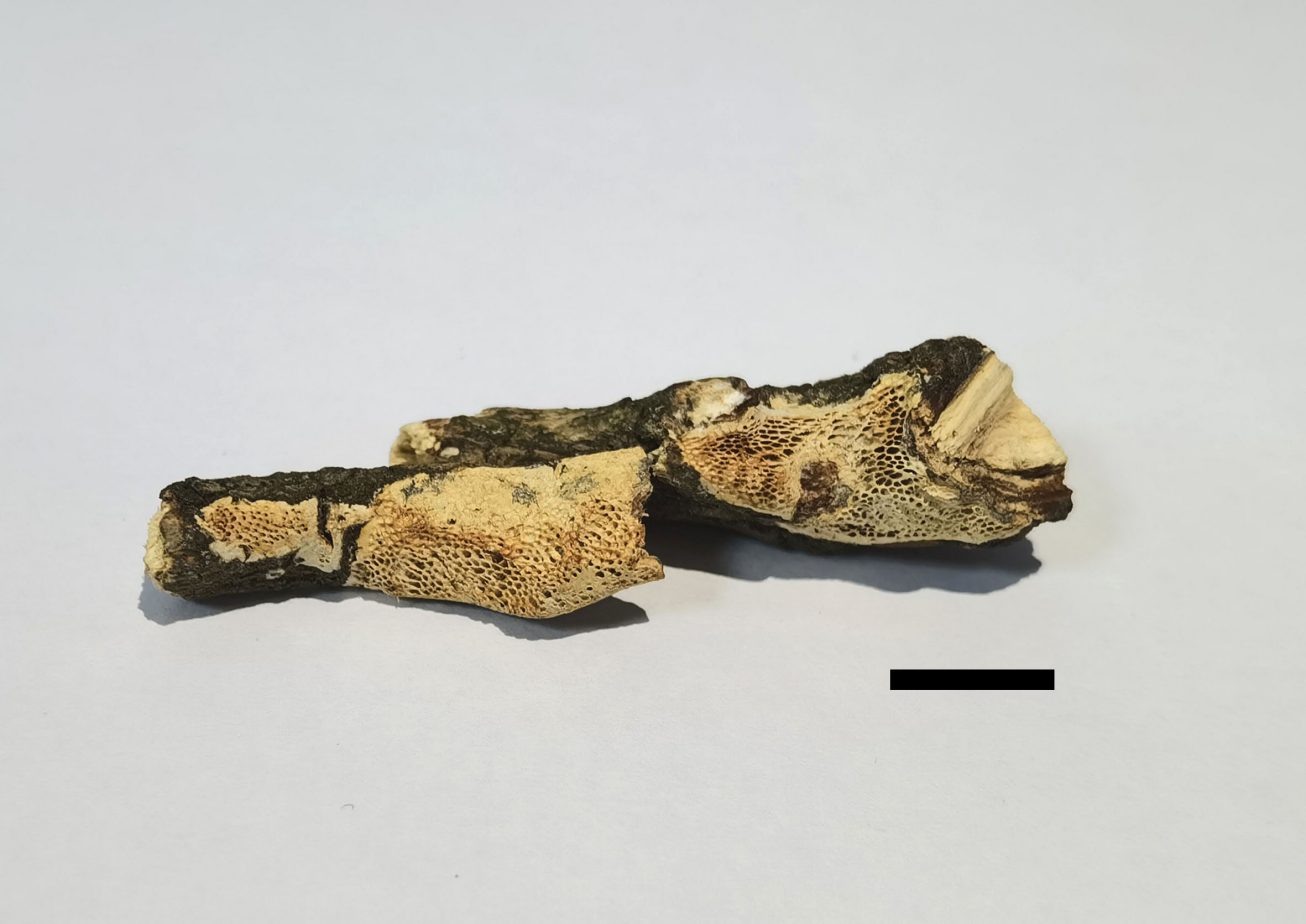
Basidiomata of *Haploporus ecuadorensis* (Holotype, JV1906/C10-J). Scale bar = 1.0 cm.

**Figure 3:**
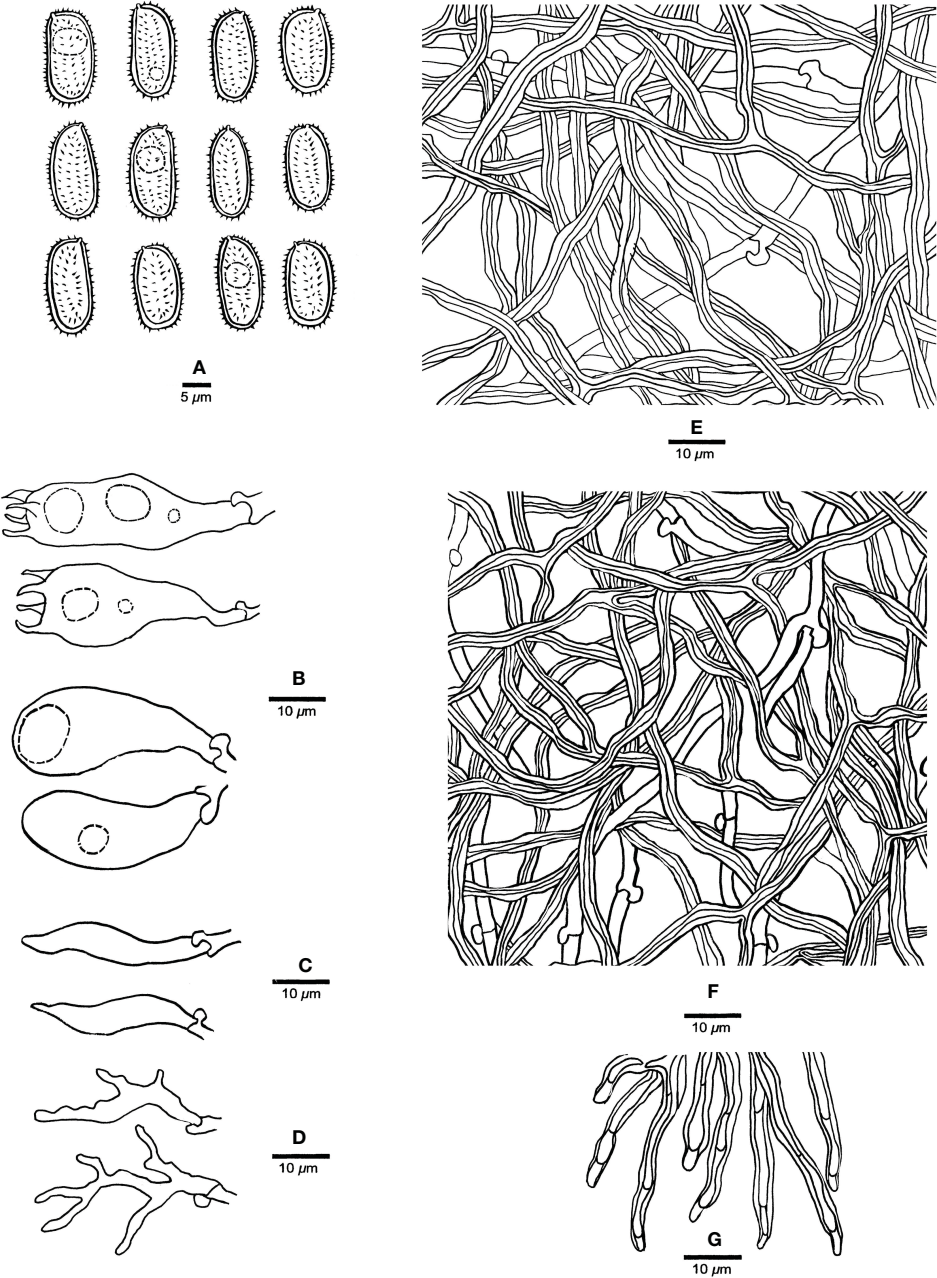
Microscopic characteristics of *Haploporus ecuadorensis* (Holotype, JV1906/C10-J). **(A)** Basidiospores. **(B)** Basidioles and basidia. **(C)** Cystidioles. **(D)** Dendrohyphidia. **(E)** Hyphae from subiculum. **(F)** Hyphae from tube trama. **(G)** Dissepiment hyphae. Scale bars: a = 5 μm, b–e = 10 μm.

***Haploporus ecuadorensis*** Y.C. Dai, Meng Zhou, & Vlasák, sp. nov. (**Figures 2** , **3**)

MycoBank: MB847499

Etymology: *ecuadorensis* (Lat.): Refers to the occurrence of the species in Ecuador.

Type: Ecuador, Pichincha, Vicodin svah Volcán Pasochoa, on dead angiosperm branch, June 2019 JV1906/C10-J (Holotype PRM, isotypes BJFC 032988 and JV).

Basidiomata resupinate, annual, inseparable from the substrate, more or less corky when dry, up to 5 cm long, 1.5 cm wide, and 1.5 mm thick at the center. Hymenophore pinkish buff to honey yellow when dry, without distinct margin; pores angular to round, 2–4 per mm; dissepiments thick, entire. Subiculum paler than tubes, more or less corky, up to 0.5 mm thick. Tubes olivaceous buff, hard corky, up to 1.0 mm long.

Hyphal system dimitic; generative hyphae with clamp connections; skeletal hyphae thick-walled, frequently branched, neither amyloid nor dextrinoid in Melzer’s reagent, cyanophilous in Cotton Blue; tissues unchanged in 5% potassium hydroxide.

Subicular generative hyphae hyaline, thin-walled, sometimes branched, 2.2–3.3 µm in diameter; skeletal hyphae dominant, with a narrow to wide lumen, usually branched, flexuous, interwoven, 3–5.2 µm in diameter.

Tube tramal generative hyphae hyaline, thin-walled, usually branched, 1.6–3.2 µm in diameter; skeletal hyphae dominant, with a narrow lumen, usually branched, strongly flexuous, distinctly interwoven, 2.2–4 µm in diameter. Cystidioles fusiform with a sharp tip, thin-walled, hyaline, 23–34 × 4–6 µm. Basidia more or less capitate to pyriform, with four sterigmata, sometimes with a few small guttules, 40–45 × 13–15 µm, clamped at the base; basidioles capitate to pyriform, almost the same size as basidia. Dissepiment hyphae thick-walled with one or two simple septa. Dendrohyphidia present among hymenium, thin-walled, hyaline. Large and irregularly shaped crystals sometimes present among trama.

Basidiospores oblong to ellipsoid, thick-walled, tuberculate, hyaline, some with a guttule, neither amyloid nor dextrinoid in Melzer’s reagent, cyanophilous in Cotton Blue, (14.3–)14.9–17.9(–19) × (6.5–)6.9–8.8(–9) µm, arithmetic average length *L* = 15.94 µm, arithmetic average width *W* = 7.67 µm, and *L*/*W* ratio *Q* = 2.07 (*n* = 30/1).

Distribution and ecology: *Haploporus ecuadorensis* is distributed in tropical areas of Pichincha, Ecuador; it grows on dead angiosperm branch and causes a white rot.

**Figure 4:**
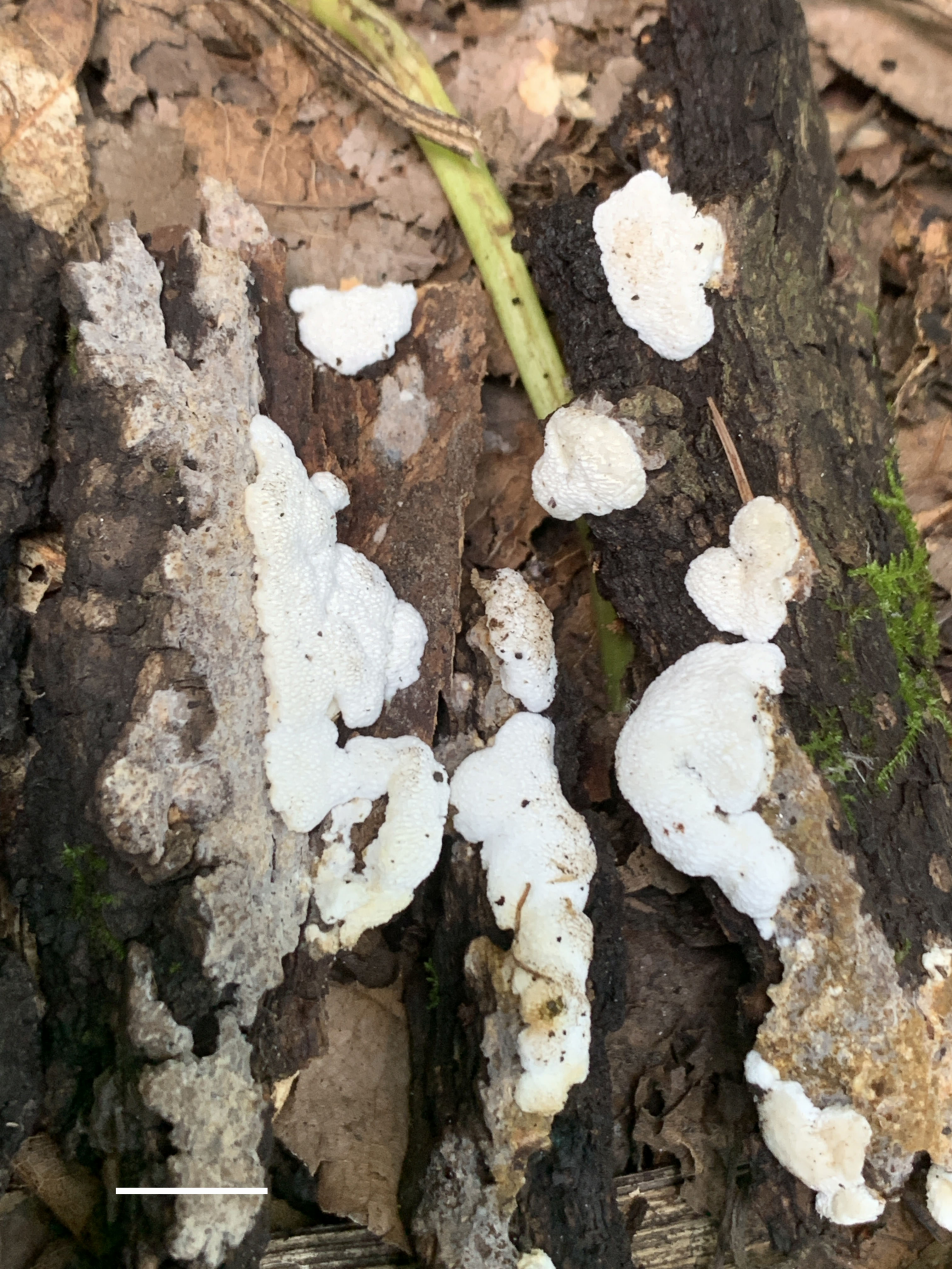
Basidiomata of *Haploporus monomitica* (Holotype, Dai 24446). Scale bar = 1 cm. Photo by Yu-Cheng Dai.

**Figure 5:**
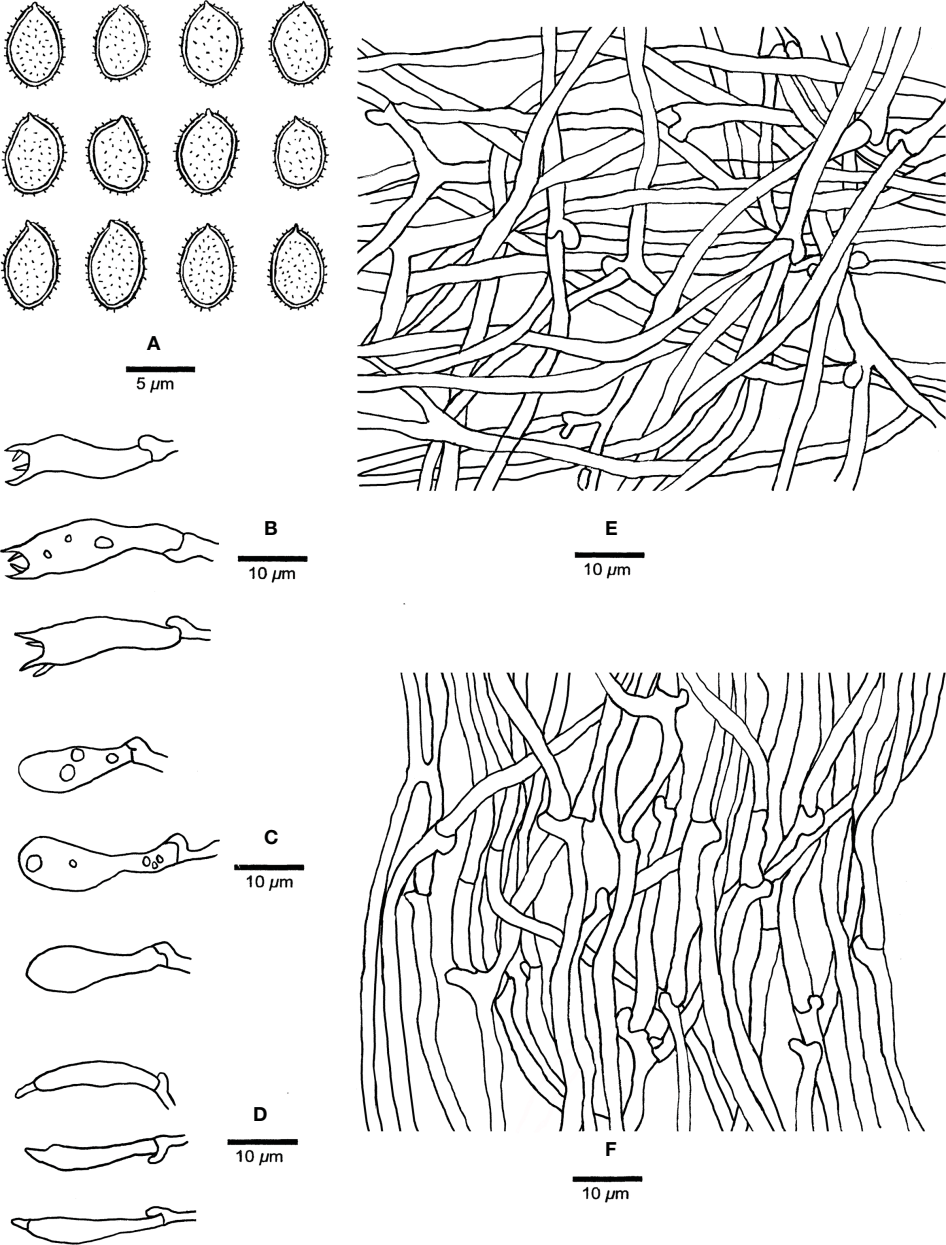
Microscopic structures of *Haploporus monomitica* (Holotype Dai 24446). **(A)** Basidiospores. **(B)** Basidia. **(C)** Basidioles. **(D)** Cystidioles. **(E)** Hyphae from subiculum. **(F)** Hyphae from trama.

***Haploporus monomitica*** Y.C. Dai, sp. nov. (**Figures 4**, **5**)

MycoBank: MB838450

Etymology: *monomitica* (Lat.): refers to the species having a monomitic hyphal system.

Type: China, Beijing, Mentougou, Xiaolongmen National Forest Park, on fallen trunk of *Quercus* sp., 30 August 2022, Yu-Cheng Dai, Dai 24446 (Holotype BJFC 038932).

Basidiomata annual, resupinate, difficult to separate from the substrate, soft and white when fresh, become soft corky to fragile and white to cream when dry, up to 3 cm long, 1 cm wide, and 1 mm thick at the center. Sterile margin distinct, white, cottony, up to 1 mm; pores round to angular, 3–4 per mm; dissepiments thick, entire. Subiculum white, soft corky, up to 0.2 mm thick. Tubes concolorous with pores, fragile, up to 0.8 mm long.

Hyphal system monomitic; generative hyphae bearing clamp connections, hyaline, thin-walled, frequently branched, neither amyloid nor dextrinoid in Melzer’s reagent, cyanophilous in Cotton Blue; tissues unchanging in 5% potassium hydroxide.

Subicular generative hyphae hyaline, thin-walled, frequently branched, flexuous, interwoven, 2–3.3 µm in diameter.

Tube tramal generative hyphae hyaline, thin-walled, frequently branched, flexuous, interwoven, 2–3 µm in diameter. Cystidia absent; cystidioles present, clavate to fusiform, hyaline, thin-walled, 17–25 × 3–5 µm. Basidia clavate with 4-sterigmata and a basal clamp connection, 15–32 × 6–9 µm; basidioles pyriform, slightly smaller than basidia. Dendrohyphidia absent.

Basidiospores broadly ellipsoid, hyaline, thick-walled with echinulate ornamentation, dextrinoid in Melzer’s reagent, cyanophilous in Cotton Blue, (4.2–)4.9–6.5 × (3.0–)3.2–4.8(–5.0) µm, arithmetic average length *L* = 5.37 µm, arithmetic average width *W* = 3.90 µm, and *L*/*W* ratio *Q* = 1.32–1.43 (n =90/3).

Additional materials studied: China, Beijing, Mentougou, Xiaolongmen National Forest Park, on fallen trunk of *Quercus* sp., 30 August 2022, Yu-Cheng Dai, Dai 24429, Dai 24451.

Distribution and ecology: *Haploporus monomitica* is distributed in temperate area of Beijing, China; it grows on fallen trunk of *Quercus*, and causes a white rot.

## Discussion

In a combined ITS + LSU + mtSSU dataset-based phylogeny (**Figure 1**) *Haploporus ecuadorensis* forms an independent lineage that is closely related to *H. grandisporus* Decock, *H. eichelbaumii* (Henn.) Decock and *H.* sp. (Decock et al., 2021). Morphologically, *H. eichelbaumii* is different from *H. ecuadorensis* in that it has smaller basidiospores (11–14 × 5.3–6.5 µm *vs*. 14.9–17.9 × 6.9–8.8 µm; Decock et al., 2021). *H. grandisporu*s is readily distinguished from *H. ecuadorensis* by larger pores (1.5–2.5 per mm *vs*. 2–4 per mm) and narrower basidiospores (14–17.5 × 6–7.3 µm *vs*. 14.9–17.9 × 6.9–8.8 µm; Decock et al., 2021). *Haploporus* sp. From Malawi is also an independent lineage within the *Haploporus* clade in a previous study (Decock et al., 2021). This taxon differs from *H. ecuadorensis* in that it has distinctly smaller pores (4–5 *vs*. 2–4 per mm Decock et al., 2021). In addition, there are more than 2% nucleotide difference in the ITS sequences between *Haploporus* sp. and *H. ecuadorensis*.

*Haploporus ecuadorensis*, *H. crassus*, *H. pirongia*, and *H. septatus* share thick-walled dissepiment hyphae with a simple septum or a few septa. *Haploporuscrassus* can be differentiated from *H. ecuadorensis* by its thick-walled basidia, the ventricose cystidioles occasionally with a simple septum, and the absence of dendrohyphidia (Zhou et al., 2019). *Haploporus pirongia* is distinguished from *H. ecuadorensis* by smaller basidiospores (11–14 × 5.2–7 µm *vs*. 14.9–17.9 × 6.9–8.8 µm; Zhou et al., 2019). *Haploporus septatus* is different from *H. ecuadorensis* in that it has dextrinoid skeletal hyphae in Melzer’s reagent and smaller pores and basidiospores (5–6 per mm *vs*. 2–4 per mm, 8.5–11 × 5–6 μm *vs*. 14.9–17.9 × 6.9–8.8 µm; Shen et al., 2016).

*Haploporus longisporus* resembles *H. ecuadorensis* in terms of resupinate basidiomata, similar pore dimension (2–3 per mm *vs*. 2–4 per mm), non-dextrinoid skeletal hyphae in Melzer’s reagent, and the presence of dendrohyphidia and cystidioles. Although both species have an overlapping distribution in Ecuador, *H. longisporus* is readily distinguished from *H. ecuadorensis* by bigger basidiospores (18.2–22 × 7–9 µm *vs*. 14.9–17.9 × 6.9–8.8 µm; Zhou et al., 2021).

*Haploporus gilbertsonii* was described from the USA recently (Zhou et al., 2021). It is similar to *H. ecuadorensis* in terms of resupinate basidiomata, similar pore dimension (2–3 per mm *vs*. 2–4 per mm; Zhou et al., 2019), non-dextrinoid skeletal hyphae in Melzer’s reagent, and the presence of cystidioles, but the former differs from the latter by the absence of dendrohyphidia and smaller basidiospores (12–15 × 6–8 µm *vs*. 14.9–17.9 × 6.9–8.8 µm; Zhou et al., 2021).

Our phylogeny shows that *Haploporus monomitica* forms a sister group to *H. odorus* with strong support (BP: 91%, MP: 84%, and BPP 1.0). However, *H. odorus* has pileate basidiomata with a strong fragrant odor, a dimitic hyphae system, non-dextrinoid or very weakly dextrinoid basidiospores, and grows exclusively on *Salix* (Niemelä, 2005; Zhou et al., 2021). Moreover, in Siberia and North America, the fungus grows on another member of the Salicaceae family, *Populus tremula* (Zmitrovich et al., 2019).

The dimitic or trimitic hyphal structure was mentioned in the previous definition of *Haploporus* (Ryvarden & Melo, 2014; Shen et al., 2016; Zhou et al., 2019; Decock et al., 2021; Zhou et al., 2021); however, a monomitic hyphal system is found in the new species *Haploporus monomitica*, and phylogenetically, it is nested in *Haploporus*. Therefore, the updated definition of the genus is as follows: basidiomata annual to perennial, resupinate to pileate, hyphal system monomitic, dimitic to trimitic with clamped generative hyphae, cyanophilous skeletal hyphae, thick-walled, cyanophilous, and ornamented basidiospores, and causing a white rot.

Like other genera of wood-decaying fungi having a rich diversity of species in tropical areas (Wu et al., 2017; Cui et al., 2019; Wu et al., 2020; Dai et al., 2021; Guan & Zhao, 2021; Wang et al., 2021; Wu et al., 2021; Ma et al., 2022), our result shows that a high diversity of *Haploporus* exists in neotropical areas.

## Key to species of *Haploporus*

1. Hyphal system monomitic*.................................H. monomitica*
1. Hyphal system dimitic to trimitic.............................................2
2. Basidiospores < 8 µm long..........................................................3
2. Basidiospores > 8 µm long..........................................................6
3. Pores 7–9 per mm.........................................................................4
3. Pores < 6 per mm..........................................................................5
4. Cystidioles absent...................................................*H. nanosporus*
4. Cystidioles present................................................*H. microsporus*
5. Pores 1–3 per mm; skeletal hyphae strongly dextrinoid...............................................................*H. brasiliensis*
5. Pores 4–5 per mm; skeletal hyphae weakly dextrinoid...............................................................*H. odorus*
6. Basidiomata annual to perennial................................................7
6. Basidiomata annual.......................................................................9
7. Skeletal hyphae dextrinoid...................................*H. srilankensis*
7. Skeletal hyphae non-dextrinoid..................................................8
8. Basidiospores cylindrical...............................................*H. thindii*
8. Basidiospores oblong ellipsoid to ellipsoid..................................
*...........................................................................H. subtrameteus*
9. Hyphal system trimitic...............................................................10
9. Hyphal system dimitic................................................................12
10. Skeletal hyphae dextrinoid...............................*H. tuberculosus*
10. Skeletal hyphae non-dextrinoid..............................................11
11. Basidiospores ovoid to ellipsoid...........................*H. alabamae*
11. Basidiospores oblong-ellipsoid to cylindrical.......*H. pirongia*
12. Cystidioles absent......................................................................13
12. Cystidioles present....................................................................15
13. Basidiomata pileate....................................................*H. pileatus*
13. Basidiomata resupinate............................................................14
14. Pores 4–5 per mm, basidiospores cylindrical, 10–11.5 × 4.5–5 µm..................................*H. cylindrosporus*
14. Pores 1.5–4 per mm, basidiospores ellipsoid to oblong, 10–15 × 5–6.8 µm.................................*H. eichelbaumii*
15. Dendrohyphidia present..........................................................16
15. Dendrohyphidia absent............................................................20
16. Pores 5–7 per mm.........................................................*H. bicolor*
16. Pores < 4 per mm.......................................................................17
17. Basidiospores cylindrical..........................................................18
17. Basidiospores ellipsoid to oblong...........................................19
18. Basidiospores 18.2–22 × 7–9 µm.......................*H. longisporus*
18. Basidiospores 13–15 × 5–6 µm...........................*H. papyraceus*
19. Hyphal system trimitic, skeletal hyphae dextrinoid.............................*H. grandisporu*s
19. Hyphal system dimitic, skeletal hyphae non-dextrinoid.......................................................................*H. ecuadorensis*
20. Pores > 3 per mm.......................................................................21
20. Pores < 3 per mm.......................................................................25
21. Pores 5–6 per mm......................................................*H. septatus*
21. Pores 3–5 per mm......................................................................22
22. Skeletal hyphae non-dextrinoid................................*H. crassus*
22. Skeletal hyphae dextrinoid.......................................................23
23. Cystidioles without septum............................*H. angustisporus*
23. Cystidioles with a simple septum............................................24
24. Basidiospores 9–10.8 × 3.8–5 µm.........................*H. punctatus*
24. Basidiospores 9–12 × 5.5–8 µm...................*H. subpapyraceus*
25. Basidiospores 9–10 µm wide.................................*H. latisporus*
25. Basidiospores < 9 µm wide.......................................................26
26. Basidiospores 12–15 × 6–8 µm...........................*H. gilbertsonii*
26. Basidiospores 8.5–11.5 × 4.5–6.5 µm..*...............H. nepalensis*

## Data availability statement

The datasets presented in this study can be found in GenBank Database. The names of the accession numbers can be found in the **Table 1**.

## Author contributions

Y-CD, L-SB, HZ and JV collected specimens. X-WM, L-SB, MZ and HZ did the drawings, DNA sequencing, and data analyses, and drafted the paper. JV and Y-CD did the morphological descriptions and acquired funding. All authors contributed to the article and approved the submitted version.
